# Dysregulation of H/ACA ribonucleoprotein components in chronic lymphocytic leukemia

**DOI:** 10.1371/journal.pone.0179883

**Published:** 2017-06-30

**Authors:** Patricia Carolina Dos Santos, Julieta Panero, Carmen Stanganelli, Virginia Palau Nagore, Flavia Stella, Raimundo Bezares, Irma Slavutsky

**Affiliations:** 1 Laboratorio de Genética de Neoplasias Linfoides, Instituto de Medicina Experimental, CONICET-Academia Nacional de Medicina, Buenos Aires, Argentina; 2 División Patología Molecular, Instituto de Investigaciones Hematológicas-Academia Nacional de Medicina, Buenos Aires, Argentina; 3 Servicio de Hematología, Hospital Teodoro Álvarez, Buenos Aires, Argentina; University of Newcastle, UNITED KINGDOM

## Abstract

Telomeres are protective repeats of TTAGGG sequences located at the end of human chromosomes. They are essential to maintain chromosomal integrity and genome stability. Telomerase is a ribonucleoprotein complex containing an internal RNA template (hTR) and a catalytic subunit (hTERT). The human hTR gene consists of three major domains; among them the H/ACA domain is essential for telomere biogenesis. H/ACA ribonucleoprotein (RNP) complex is composed of four evolutionary conserved proteins, including dyskerin (encoded by *DKC1* gene), NOP10, NHP2 and GAR1. In this study, we have evaluated the expression profile of the H/ACA RNP complex genes: *DKC1*, *NOP10*, *NHP2* and *GAR1*, as well as *hTERT* and *hTR* mRNA levels, in patients with chronic lymphocytic leukemia (CLL). Results were correlated with the number and type of genetic alteration detected by conventional cytogenetics and FISH (fluorescence in situ hybridization), *IGHV* (immunoglobulin heavy chain variable region) mutational status, telomere length (TL) and clinico-pathological characteristics of patients. Our results showed significant decreased expression of *GAR1*, *NOP10*, *DKC1* and *hTR*, as well as increased mRNA levels of *hTERT* in patients compared to controls (p≤0.04). A positive correlation between the expression of *GAR1*-*NHP2*, *GAR1*-*NOP10*, and *NOP10*-*NHP2* (p<0.0001), were observed. The analysis taking into account prognostic factors showed a significant increased expression of *hTERT* gene in unmutated-IGHV cases compared to mutated-CLL patients (p = 0.0185). The comparisons among FISH groups exhibited increased expression of *DKC1* in cases with two or more alterations with respect to no abnormalities, trisomy 12 and del13q14, and of *NHP2* and *NOP10* compared to those with del13q14 (p = 0.03). The analysis according to TL showed a significant increased expression of *hTERT* (p = 0.0074) and *DKC1* (p = 0.0036) in patients with short telomeres compared to those with long TL. No association between gene expression and clinical parameters was found. Our results suggest a role for these telomere associated genes in genomic instability and telomere dysfunction in CLL.

## Introduction

Telomeres are protective repeats of TTAGGG sequences located at the end of chromosomes [1]. One important function of the telomere is to prevent end-to-end joining of chromosomes, which otherwise could lead to the accumulation of chromosomal breaks during mitosis. Due to the end-replication problem, telomeres become progressively shorter with repeated cell division. Telomere shortening leads to telomere dysfunction and elicitation of a DNA damage response. Moreover, it has implications in oncogenesis and cellular aging due to limited cellular proliferation below a certain critical telomere length (TL) [2].

TL is maintained by the enzymatic complex telomerase. This enzyme participates in the formation and maintenance of telomeres in normal progenitor cells [3] and is active in most of tumors and immortal cell lines, endowing cancer cells with an infinite replicative capacity [4, 5]. Telomerase consists of two core components: a catalytic subunit, human telomerase reverse transcriptase (hTERT) with reverse transcriptase activity and a RNA component, human telomerase RNA component (hTR) that serves as a template for the elongation of telomeres. As described in detail previously [6] human hTR extends for 451 nucleotides, the last 240 at the 3´end form a consensus H/ACA two hairpin structure while the 5' half folds into a pseudoknot containing the template for the reverse transcriptase. While it is not needed for telomerase activity *in vitro*, the H/ACA domain of hTR is required for its *in vivo* accumulation and stability [6, 7]. Telomerase activity requires additional ribonucleoprotein (RNP) factors that associate with hTERT and hTR to form the holoenzyme. Thus, H/ACA RNPs consist of four evolutionarily conserved proteins, Dyskerin [encoded by the gene *DKC1* (*Dyskerin Pseudouridine Synthase 1*)], NHP2 (*NHP2 ribonucleoprotein*), NOP10 (*NP10 ribonucleoprotein*), and GAR1 (*GAR1 ribonucleoprotein*), and a function-specifying, noncoding H/ACA RNAs [8, 9]. All these RNPs are concentrated in nucleoli and Cajal bodies of mammalian cells, reflecting the location of H/ACA RNPs [10]. In reference to its functions, H/ACA RNPs contribute to telomerase assembly and stabilization, posttranscriptional processing of nascent ribosomal RNA and pre-mRNA splicing [6]. Dyskerin, NHP2 and NOP10 are essential for the cellular accumulation of the H/ACA-motif RNAs, while GAR1 binds only to Dyskerin and is necessary for the nucleolar localization and function of the RNP complex [11, 12]. Particularly, Dyskerin functions as a pseudouridine synthase and is involved in post-transcriptional modifications of rRNA [13]. In addition, Dyskerin is part of the telomerase complex and is required for the correct activity of the enzyme, which directly binds to and stabilizes hTR within the complex [14], playing a role in the maintenance of telomere integrity. Point mutations in *DKC1* gene cause the X-linked form of dyskeratosis congenital, a disease with increased predisposition to cancer.

Chronic lymphocytic leukemia (CLL) is the most frequent leukemia in adults in Western countries; it is a heterogeneous disease with variable clinical presentation and evolution [15]. At the molecular level, two major subtypes can be distinguished, mutated (M) and unmutated (UM), characterized respectively by a high or low number of somatic hypermutations in the variable region of immunoglobulin genes and different clinical evolution [16, 17]. Cytogenetic and FISH (*fluorescence in situ hybridization*) studies have also proved to be important tools in the biologic characterization of this disease, allowing the identification of distinct cytogenetic risk groups and providing information for clinical outcome [18, 19]. In addition, short telomeres have been associated with genetic complexity and a high risk of genomic aberrations [20–23], and ultimately implicated in disease outcome [21, 22, 24]. Increased telomerase expression and activity were also observed in CLL patients [25, 26]. However, data concerning the participation of H/ACA RNPs in telomere dysfunction in CLL are lacking. In the present study, we investigated the expression profile of *DKC1*, *NHP2*, *NOP10* and *GAR1*, in association with *hTR* and *hTERT* mRNA levels. Results were correlated with the number and type of genetic alterations detected by conventional cytogenetics and FISH, the mutational status of *IGHV* (*immunoglobulin heavy chain variable region*), TL and clinico-pathological characteristics of patients.

## Materials and methods

### Patients

This study was based on a retrospective series of 71 CLL patients at diagnosis (31 females; mean age 67 years, range: 44–88 years) derived to our Laboratory from 2006 to 2016, and from whom RNA samples were available. Patients were diagnosed according to the National Cancer Institute-Working Group CLL criteria [27]. Rai stages [28] were available for 54 cases with the following distribution: 0: 19 (35.2%); I–II: 30 (55.5%); and III–IV: 5 (9.3%). Table 1 shows clinical and biological characteristics of patients. In addition, 21 normal controls (NC) (without a medical history of cancer) (10 females; mean age 62 years, range: 40–89 years), were properly selected to this study. The study was approved by the Ethics Committee of the Institutes of the National Academy of Medicine of Buenos Aires (Approval number: T.I.N° 12042/15/X). All patients provided their written informed consent.

**Table 1:** Clinical and biological characteristics of CLL patients.

Characteristics	
N° of patients (n)	71
Sex F/M	31/41
Mean age (years) (range)	67 (44–88)
Clinical stages (%)	
Rai 0	19 (35.2)
Rai I–II	30 (55.5)
Rai III–IV	5 (9.3)
Mean WBC count (×10^9^/L) (range)	41.4 (9.1–210)
Mean lymphocyte (%) (range)	76.1 (15.5–97)
Mean Plt count (×10^9^/L) (range)	212.3 (67–900)
Mean Hb (g/dL) (range)	12.5 (7–15.9)
Mean LDH (UI/L) (range)	380 (128–647)
Mean β_2_M (μg/mL) (range)	2.77 (0.37–8)

F: female, M: male, WBC: white blood cells, Plt: platelets, Hb: hemoglobin, LDH: lactate dehidrogenase, β_2_M: β2-microglobulin.

### RNA extraction and reverse transcription (RT)

Total RNA was extracted from mononuclear cells isolated on a Ficoll-Paque Plus (GE Healthcare Bio-Sciences) density gradient of peripheral blood (PB) samples of patients, normal controls (NC) and the K-562 cell line, using the Trizol reagent. K-562 cell line was obtained from the Laboratory of Genetics in Hematology, Institute of Experimental Medicine, CONICET-National Academy of Medicine (Buenos Aires, Argentina). The complementary DNA (cDNA) synthesis was carried out by using Moloney Murine Leukemia Virus reverse transcriptase (MMLV) and random primer (Promega). The cDNA synthesis was performed in a final volume of 20 μl, containing 1μg of the total RNA, for 10 minutes at 70°C, for 60 minutes at 37°C and 10 minutes at 95°C to inactive the enzyme. cDNA was stored at -20°C until use.

### Gene expression analysis by quantitative real-time RT-PCR

The analysis of four genes of the RNP complex *GAR1*, *NHP2*, *NOP10* and *DKC1*, as well as both telomerase units, *hTR* and *hTERT*, were performed using real-time quantitative PCR (qPCR) on a Rotor GENE Q (Qiagen), based on SybrGreen methodology. Primer sequences for *hTR* and *hTERT* were previously described [29, 30]. For the remaining genes, primers were designed for this work and detailed in Table 2. All PCR runs were performed in duplicate, using 2.5 μl of RT reaction, 2X SYBR^®^ Select Master Mix (Applied Biosystems^®^), 10 mM of each primer, in a 20 μl final volume.

**Table 2:** Primer sequences.

Name of *primer*	Sequence (5’– 3’)	Length (pb)	Reference
β-actin F	CCAGAGGCGTACAGGGATAG	97	[32]
β-actin R	CCAACCGCGAGAAGATGA		
hTR F	TCTAACCCTAACTGAGAAGG	126	[30]
hTR R	GTTTGCTCTAGAATGAACGG		
hTERT F	TGACACCTCACCTCACCCAC	95	[31]
hTERT R	CACTGTCTTCCGCAAGTTCAC		
NOP10 F	TTCGGACTGTGAGCCCTGATGCCTTT	70	Designed in this study
NOP10 R	TCAATCGCCACGAGAGACTGGATGCC		
NHP2 F	CTTCTGTCCATCAGTGCCAT	105	Designed in this study
NHP2 R	AGCATTTACTTTCCCCACCC		
DKC1 F	TGAAGAGAGAGATTGGGGACT	85	Designed in this study
DKC1 R	ATGGGAAGAGGGGTTAGAGG		
GAR1 F	CGGAGGTCGTGGAGGCTTT	77	Designed in this study
GAR1 R	CTCGGAAGTGGTTGCTGCTG		

F: forward; R: reverse.

The constitutive expression gene *β-Actin* was used to normalize sample-to-sample differences in cDNA input, RNA quality and RT efficiency [31]. For all targets, the thermal cycling conditions were 2 minutes at 50°C, followed by 2 minutes at 95°C; continued by 45 cycles at 95°C for 20 seconds and 60°C for 1 minute, ending with a melting curve from 50°C to 99°C. All measurements included a determination of the standards and no-template as a negative control, in which water was substituted for the cDNA. Standard curves were constructed with fivefold serial dilutions of the cDNA from the K-562 cell line.

### Cytogenetics and FISH analysis

Chromosome analyses were performed on PB lymphocytes, cultured for 96 h at 37°C in F-12 medium supplemented with 15% of fetal calf serum, stimulated with Pokeweed mitogen. Slides were prepared by conventional method. G-banding technique was used. For FISH analysis, slides were hybridized with SE 12, OLE13q14 D13S319, OLE11q22.3 ATM, and 17p13.1 TP53 DNA probes (LiVE-LEXEL, Buenos Aires, Argentina) according to the manufacturer’s protocol. Two hundred interphase nuclei were analyzed for each probe. The cut-offs for positive values (mean of normal control plus 3 standard deviations) determined from ten cytogenetically normal donors were as follows: 3%, 10%, 7.5%, and 5.5% for trisomy 12, monosomies of D13S319, ATM, and TP53, respectively.

### Absolute telomere length analysis

High-molecular weight genomic DNA was obtained from PB mononuclear cells of CLL patients and controls, separated on a Ficoll-Paque Plus (GE Healthcare Bio-Sciences, Uppsala, Sweden) density gradient. Absolute TL measurement was carried out by real-time quantitative PCR (qPCR) in a LightCycler system (Roche Diagnostics) according to Panero et al. [32]. For each DNA sample, two consecutive reactions were performed: the first one to amplify a single copy gene, *RPLP0* (ribosomal protein lateral stalk subunit P0) (12q24.2), and the second one for telomeric sequence. Briefly, both PCRs were performed in a final volume of 20 μl using 1X SYBRGreen Master Mix (Roche), 100 nmol/L of primers, and 20 ng of DNA from patients and controls. The PCR conditions were as follows: 95°C 10 min, followed by 45 cycles of 95°C 15 s, 60°C 1 min. The melting curve was performed with 1 cycle of 95°C 20 s, 50°C 15 s, and 98°C with a temperature ramp of 0.1°C/s. All samples were analyzed in duplicate. Absolute TL (kb/diploid genome) values were calculated as previously described [33]. The conversion of absolute TL values into the equivalent Southern blot TL values (kb) was done by using the regression formula: y = 0.0746x + 0.5285, where x = Log [TL (qPCR)] and y = Log [TL (TRF)] as was previously explained [243].

### *IGHV* mutational status

The *IGHV* gene sequences were determined as previously described [34]. Briefly, amplification of *IGHV* regions by polymerase chain reaction was performed on cDNA by using VH framework region 1 consensus family specific primers (VH1-VH6) and JH primers. When amplifications of these primers were unsuccessful, an alternative set of primers that anneal to sequences in the leader region (LH1-LH6) and one antisense Cμ-primer were used. Thermal cycling conditions were 3 minutes at 93°C, followed by 33 cycles at 94°C for 30 seconds, 62°C for 30 seconds, 72°C for 30 seconds, elongation at 72°C for 7 minutes, and a final step at 4°C for 10 minutes. Polymerase chain reaction products were purified in 2% agarose gels, sequenced bidirectionally, and analyzed on an automated DNA sequence analyzer (377 ABI Prism, PE biosystem, Foster City, CA). Sequence data were analyzed by using IgBLAST (*immunoglobulin* BLAST) (http://www.ncbi.nlm.nih.gov/igblast) and the ImMunoGeneTics database (IMGT) (http://imgt.cines.fr). *IGHV* sequences with <98% homology with respect to the germ line counterpart were considered as M, whereas those with homology of 98% or higher were classified as UM [17, 18].

### Statistical analysis

Statistical analysis was performed using GraphPad Prism Version 5.0 (2008). Comparisons of data from patients and controls were performed using the Mann-Whitney test meanwhile Kruskal-Wallis test was used for comparisons among subgroups. Groupwise comparison of the distribution of clinical and laboratory variables was performed with the Student t test (for quantitative variables) and the χ2 or Fisher’s exact test (for categorical variables). Correlations between gene expression and TL or clinical variables were assessed by using the Kendall’s coefficient. The cut-off point for gene expression was selected according to receiver operating characteristic (ROC) analysis. A hierarchical clustering was employed to segregate patients groups based on the qPCR expression patterns using the Heatmapper (www.heatmapper.ca). Treatment free survival (TFS), calculated from the date of diagnosis to the first CLL-specific treatment were estimated by the Kaplan-Meier method and compared with the Log-rank test. For all tests, p<0.05 was considered statistically significant.

## Results

### Expression profile of RNP complex genes and telomerase subunits

**Fig 1:**
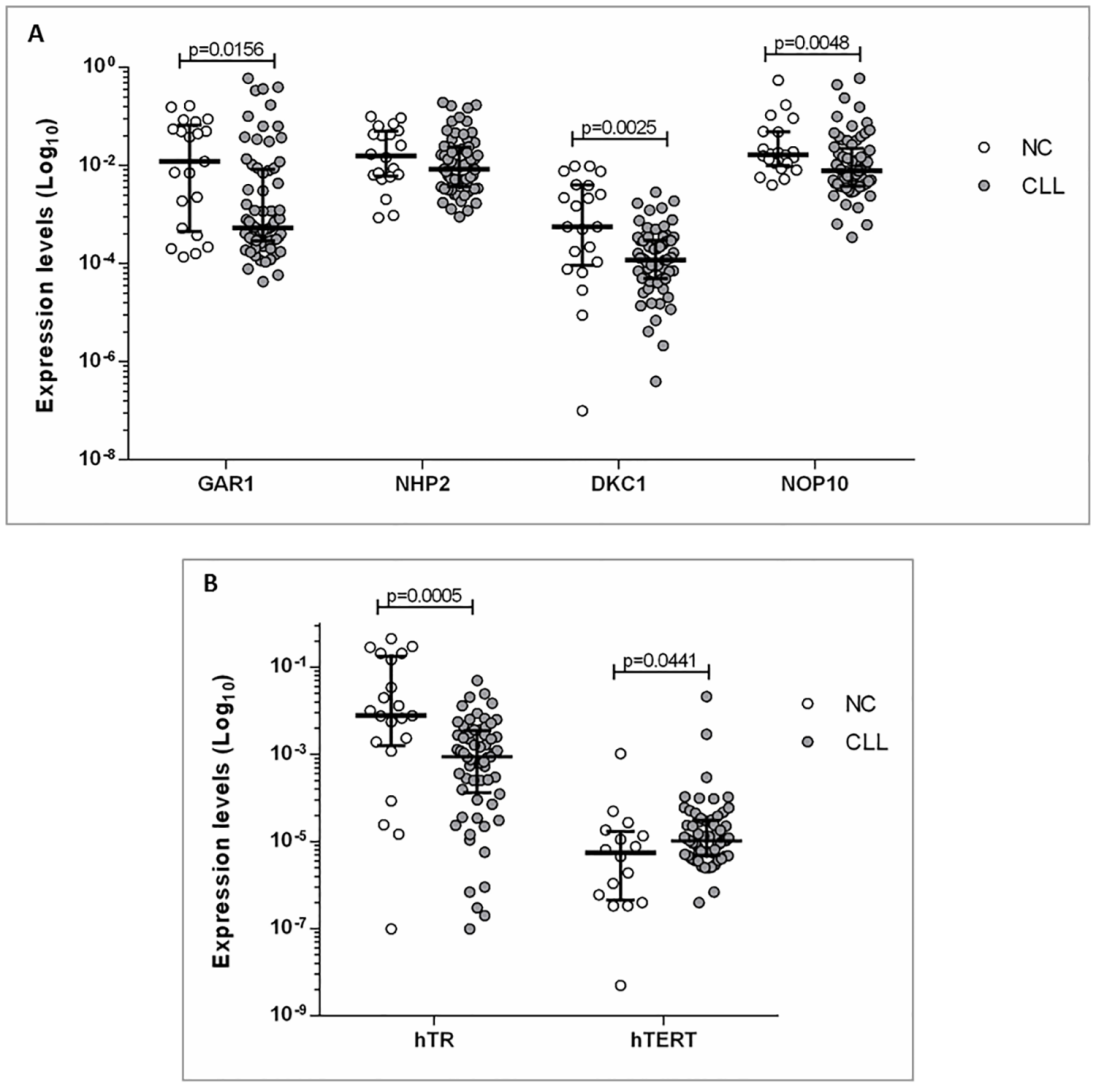
Gene expression profiles in CLL patients and normal controls (NC). (A) *GAR1*, *NHP2*, *DKC1* and *NOP10* genes. (B) *hTR* and *hTERT* genes.

The expression profile of the four RNP complex genes: *GAR1*, *NHP2*, *NOP10* and *DKC1*, as well as the two subunits of telomerase, *hTR* and *hTERT*, were evaluated in 71 CLL patients and 21 NC. The analysis showed a decreased expression of RNP complex genes in CLL patients compared to NC, with significant differences for: *GAR1* (p = 0.0156), *DKC1* (p = 0.0025) and *NOP10* (p = 0.0048). No statistical differences were found for *NHP2* mRNA expression (p = 0.182) (Fig 1A). In reference to telomerase subunits, an increase of *hTERT* (p = 0.0441) and a decrease of *hTR* (p = 0.0005) in patients compared to NC were observed (Fig 1B). In addition, a positive correlation between the expression of *GAR1*-*NHP2*, *GAR1*-*NOP10*, and *NOP10*-*NHP2* (p<0.0001) were found, indicating a strong interaction among them (Supporting information; S1 Fig). A more integrative vision of telomere-associated gene expression profiles can be observed in Supporting information (Heat map; S2 Fig).

### Correlation between expression profiles and prognostic factors in CLL

We would like to determine if the expression profile of these genes correlated with prognostic factors of relevance in CLL. Thus, we analyzed the association between transcription levels and different parameters, including the number and type of genetic alterations detected by conventional cytogenetics and FISH, the mutational status of *IGHV*, TL and clinico-pathological characteristics of patients.

**Fig 2:**
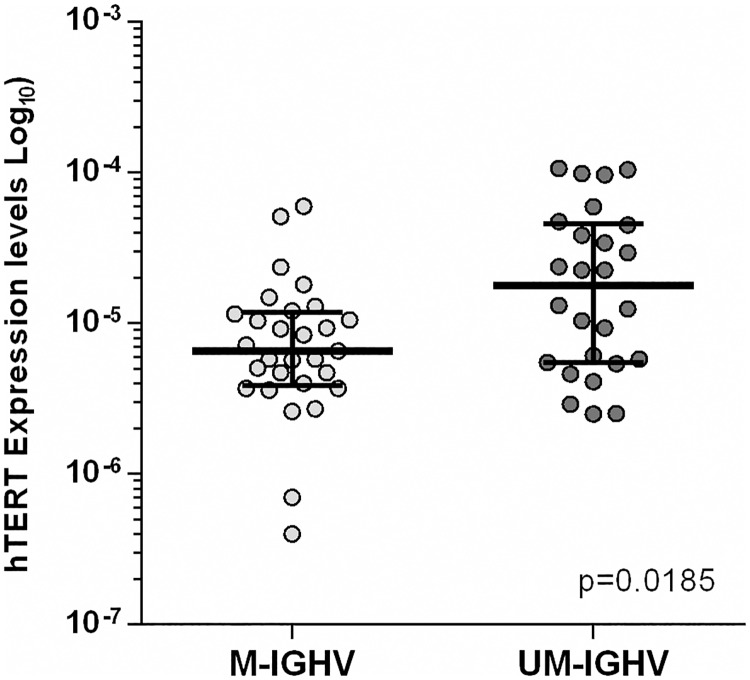
Increased expression of *hTERT* gene in unmutated (UM)-CLL patients compared to mutated (M)-CLL.

*IGHV* mutational status was evaluated in 66 cases: 34 showed M-CLL, 31 were UM-CLL and one case had a stop codon. Expression analysis of telomerase genes according to this parameter revealed significant increased expression of *hTERT* gene in UM-CLL patients (p = 0.0185) compared to M-CLL (Fig 2).

**Fig 3:**
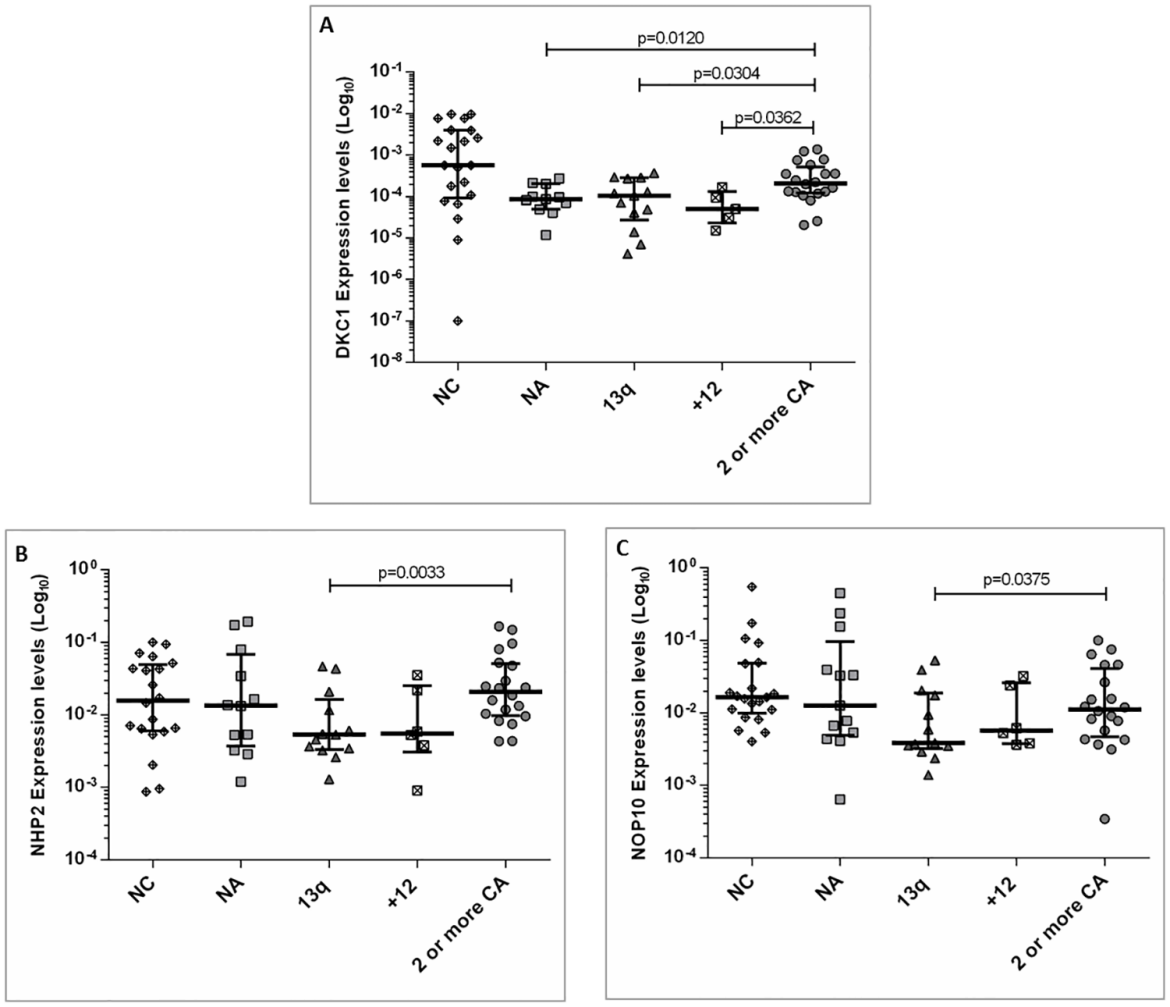
Analysis of gene expression profiles of telomere associated genes taking into account the number and type of genetic alterations in CLL patients. (A) Significant differences for *DKC1* in cases with two or more chromosome alterations (2 or more CA) with respect to no abnormalities (NA), trisomy 12 and del13q14. In addition, significant differences between normal controls (NC) and CLL patients with NA (p = 0.01134), trisomy 12 (p = 0.0274) and del13q14 (p = 0.0081) were observed; (B) Increased expression of *NHP2* in patients with 2 or more CA compared to those with del13q14; (C) Increased expression of *NOP10* in patients with 2 or more CA compared to those with del13q14. Furthermore, significant differences between NC and del13q14 (p = 0.0115) was observed.

Cytogenetic and/or FISH analysis were performed in 56 patients. Eight cases had two or more cytogenetic alterations in the karyotype, 14 showed normal karyotype and no FISH alterations, 14 had del13q14 as the only abnormality, 7 cases showed trisomy 12, and the remaining 13 had two or more FISH alterations. No differences in gene expression profiles between patients with two or more alterations by cytogenetics and FISH analysis were found, thus they were considered as a single group (2 or more CA). The analysis among groups showed significant differences for *DKC1* (p = 0.0028) and *hTR* (p = 0.0129) and a tendency for *NHP2* (p = 0.057) and *NOP10* (p = 0.068). Interestingly, when risk genetic groups were compared, an increased expression of *DKC1* in cases with 2 or more CA with respect to those with no abnormalities (NA) (p = 0.0120), trisomy 12 (p = 0.0362) and del13q14 (p = 0.0304) (Fig 3A), was observed. Moreover, overexpression of *NHP2* and *NOP10* genes in patients with 2 or more CA compared to those with del13q14 (p = 0.003 and p = 0.0375, respectively) were also found (Fig 3B and 3C).

**Fig 4:**
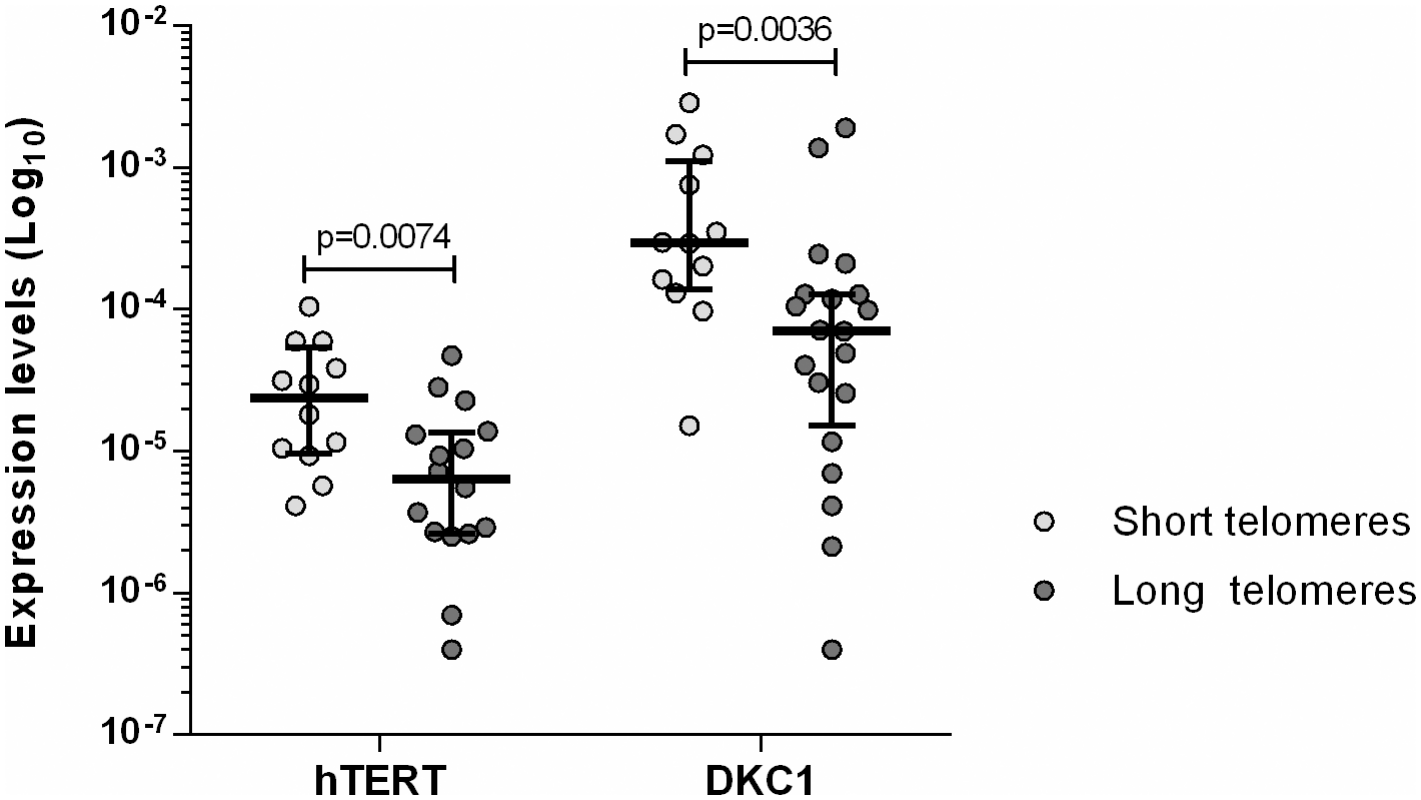
Analysis of *hTERT* and *DKC1* gene expression profiles tacking into account telomere length (TL). Significant increased expression for *hTERT* and *DKC1* in patients with short TL compared to those with long TL.

TL was measured in 32 patients of our cohort. Short telomeres were defined as below the tenth percentile of normal controls (6.63 Kb). Twelve cases had short TL and 20 showed long TL. This analysis showed a significant increased expression of *hTERT* (p = 0.0074) and *DKC1* (p = 0.0036) in patients with short telomeres compared to those with long TL. In contrast, the remaining genes did not showed significant differences (Fig 4).

Finally, the analysis taking into account clinico-pathological characteristics of patients as well as treatment-free survival and overall survival did not reveal any prognostic relevance (Supporting information; S1 Table).

## Discussion

In the present study, we have analyzed the expression profiles of four genes of the RNP complex *GAR1*, *NHP2*, *NOP10* and *DKC1*, as well as both telomerase units, *hTR* and *hTERT*, in CLL patients at diagnosis. It is important to pointing out that, to our knowledge, this is the first analysis of *GAR1*, *NHP2*, *NOP10* and *hTR* in this pathology.

Our results showed significant decreased expression *GAR1*, *NOP10*, *DKC1* and *hTR*, as well as increased mRNA levels of *hTERT* in CLL patients compared to controls, supporting the importance of changes in the expression of telomere-associated genes in this entity. There is scarce information about these genes in hematological malignancies. A report of our group [35] using microarray assay found increased expression of *NOP10*, *GAR1* and *NHP2* in myeloma cell lines and patients with plasma cell disorders, showing significant overexpression from monoclonal gammopathy of undetermined significance (MGUS) to plasma cell leukemia, indicating their association with tumor progression. On the contrary, there are some reports about *DKC1* and *hTERT* expression in CLL. In concordance with our findings, two studies found decreased expression of *DKC1* compared to controls [36, 37]. Sbarrato et al [37] suggested that the low *DKC1* expression leads to an imbalance in ribosomal proteins that could influence the response of leukemic cells to the microenvironment, and proposed a general impact of *DKC1* dysregulation on the translational machinery in CLL. Other authors suggest that *DKC1* deficiency may contribute to tumorigenesis by altering the splicing of specific mRNAs, or by modulating the level of certain snoRNAs [13]. *In vitro* experiments showed that the loss of *DKC1* function affects telomerase activity by reducing *hTR* levels, leading to premature telomere shortening that may result in chromosomal end-to-end fusions, breakage and rearrangements associated to tumor development [13].

In reference to *hTERT* expression, there are discordant results probably related with the number of patients and/or the clinical characteristics of the cohorts evaluated. Poncet et al [36] found *hTERT* down-regulation and Damle et al [38] did not observed significant differences in CLL patients compared to controls. On the contrary, Hoxha et al [3926] observed *hTERT* overexpression by microarray analysis, data that could not be validated by qRT-PCR. Interestingly, we found increased *hTERT* mRNA levels associated to UM-CLL and short TL. Conflicting results were reported about the association between *hTERT* mRNA expression and *IGHV* mutational status [21, 26, 38–40]. Our data, in concordance with those observed by Rampazzo et al [22], support the relationship between short TL, UM-*IGHV* status and increased *hTERT* expression in this pathology, as well as the importance of *hTERT* levels to maintain the replicative potential of tumor cells. In addition, a previous study of our group in patients with multiple myeloma (MM) also showed an inverse correlation between *hTERT* levels and TL, in which shortest telomeres had the highest *hTERT* expression [41]. Furthermore, it is important to pointing out that, besides maintaining the TL, *hTERT* is also involved in other cellular functions like cell survival and prevention of apoptosis [42, 43], which would be important to evaluate in CLL.

With regards to genomic aberrations, our data showed significantly increased mRNA levels of *DKC1*, *NHP2* and *NPO10* genes in CLL cases with two or more alterations. Using microarray assay, von Stedingk et al [44] found *DKC1*, *NHP2* and *GAR1* overexpression in high-stage neuroblastoma associated to poor prognosis and genomic complexity, as well as telomere dysfunction, proposing their relationship with tumor aggressiveness. More studies will be necessary to clarify the role of these genes in CLL progression. Simultaneously, we also observed increased *DKC1* expression in patients with short TL, supporting the probable involvement of this gene in progressive telomere reduction, promoting genomic instability and immortalization of cancer cells. In concordance, a previous report [45] in lung cancer cells also found a strong association between Dyskerin and short telomeres and more recently our group showed *DKC1* overexpression in MM patients with short TL [46]. In reference to *GAR1*, we did not find association with the different prognostic factors evaluated. A recent report on cell lines [47] found differential expression of this protein in response to different genotoxic agents and particularly to DNA damage response. The authors propose that this overexpression may be responsible, at least in part, to the survival and proliferations of tumor cells, suggesting that this protein may function independently of its role within the H/ACA RNP complex.

Finally, we were interested in correlating our data with clinical characteristics of patients as well as the evolution of the disease. This analysis did not reveal any prognostic relevance for the genes evaluated. The literature shows discordant results for *hTERT* expression, with authors observing strong impact of telomerase activity on overall survival [22, 25, 39] while others did not find any association [221, 276]. In addition, a recent report [37] did not find impact of *DKC1* expression on overall survival or progression free survival, but they observed that cases with low *DKC1* levels had a reduced survival following chemotherapy. There are no reports about this matter for the remaining genes.

The current study is subject to a number of limitations. Our analysis was performed in a retrospective cohort of CLL patients. Thus, as regards prognostic factors and outcome, the lack in statistical significance could be due to the limited number of patients in our series. Additionally, we have not investigated protein expression, results that would increase the significance of this study. Future analysis in larger cohorts will be useful to better define the prognostic value of these genes as well as functional modifications.

Concluding, we have observed modifications in the expression profiles of *GAR1*, *NOP10*, *DKC1*, *hTERT* and *hTR* genes in CLL patients. The correlation with prognostic factors of the disease showed associations among *DKC1*, *NHP2* and *NPO10* mRNA levels with the presence of two or more genetic alterations and of *hTERT* and *DKC1* expression with short telomere length, suggesting a role for these telomere associated genes in genomic instability and telomere dysfunction in this pathology.

## Supporting information

S1 Fig Correlation among gene expression profiles of *NHP2*, *GAR1* and *NOP10*. (TIF)

S2 Fig Heat map. Telomere-associated gene expression profiles in CLL patients.
(TIF)

S1 Table mRNA expression levels of telomere-associated genes and clinical and laboratory features. (DOCX)
