# Supplementary figures and images for: Dysregulation of H/ACA ribonucleoprotein components in chronic lymphocytic leukemia
Source: PLoS One. 2017 Jun 30;12(6):e0179883. doi: 10.1371/journal.pone.0179883 (PMC5493334; doi:10.1371/journal.pone.0179883)

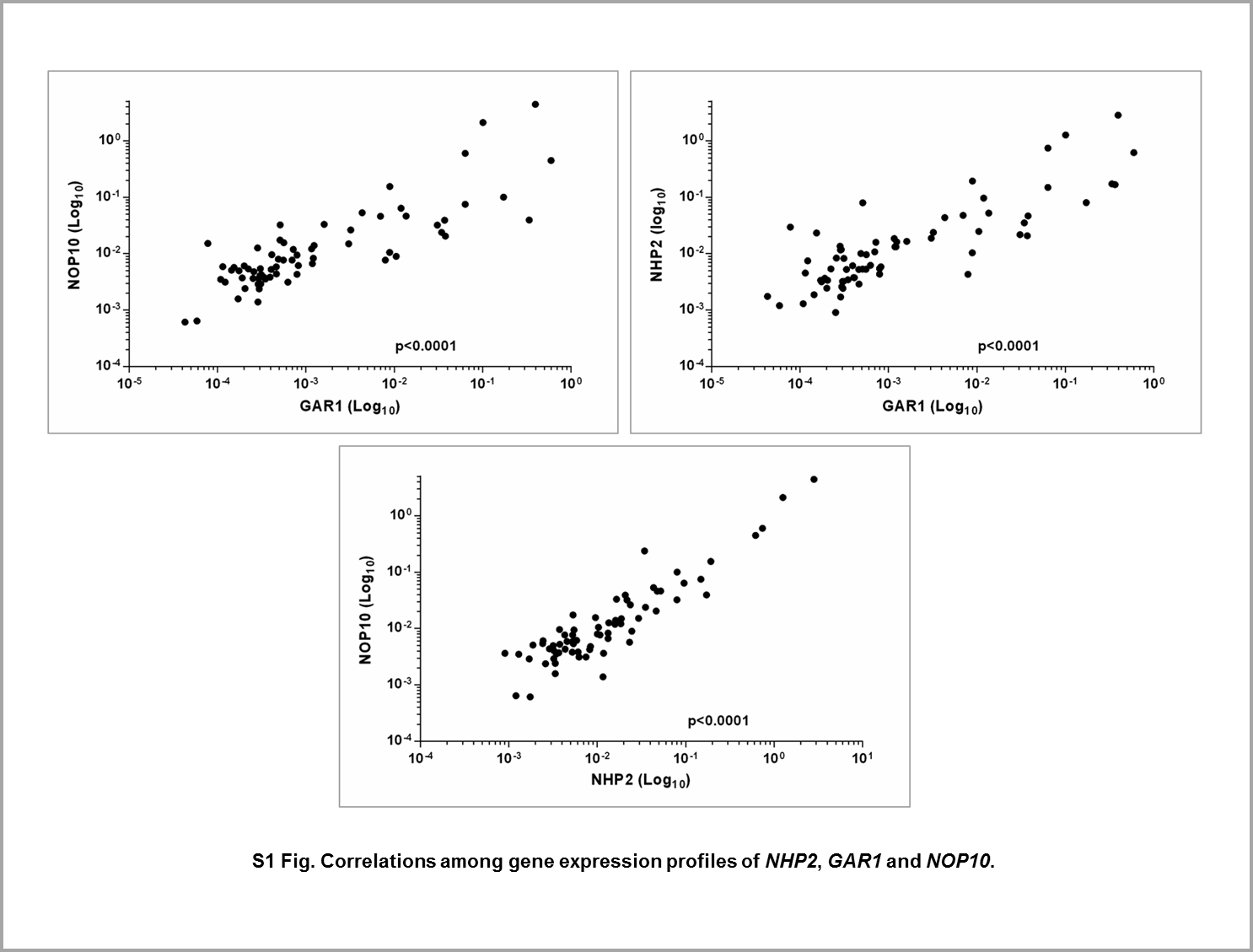

Supplement: S1 Fig — (TIF) [file pone.0179883.s001.tif]

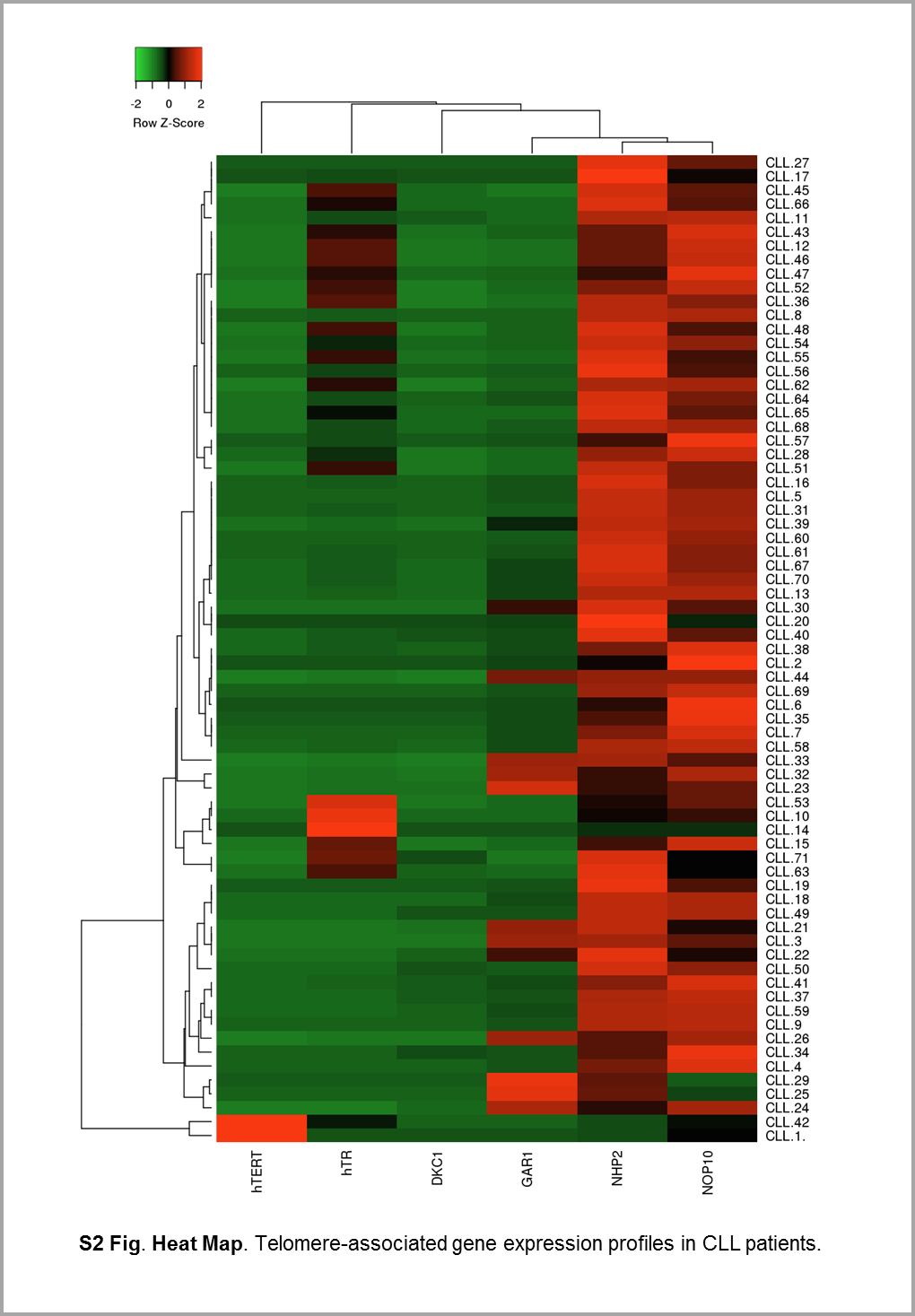

Supplement: S2 Fig — Telomere-associated gene expression profiles in CLL patients. (TIF) [file pone.0179883.s002.tif]
