# Supplementary material for: Dysregulation of H/ACA ribonucleoprotein components in chronic lymphocytic leukemia
Source: PLoS One. 2017 Jun 30;12(6):e0179883. doi: 10.1371/journal.pone.0179883 (PMC5493334; doi:10.1371/journal.pone.0179883)
Supplement: S1 Table — (DOCX) [file pone.0179883.s003.docx]

**Supporting information**

S1 Table: mRNA expression levels of telomere associated genes and clinical and laboratory features

| **Feature** | **Expression of telomeric genes (mean)** | | | | | |
| --- | --- | --- | --- | --- | --- | --- |
|  | ***hTR*** | ***hTERT*** | ***GAR1*** | ***NHP2*** | ***DKC1*** | ***NOP10*** |
| Sex |  |  |  |  |  |  |
| F | 5,42E-03 | 1,39E-05 | 3,92E-02 | 9,82E-02 | 3,47E-04 | 4,29E-02 |
| M | 1,89E-03 | 3,40E-05 | 2,87E-02 | 3,32E-02 | 2,58E-04 | 2,84E-02 |
| Rai stages |  |  |  |  |  |  |
| 0 | 4,77E-03 | 2,31E-05 | 4,31E-02 | 3,47E-02 | 3,14E-04 | 2,12E-02 |
| I/II | 4,06E-03 | 1,48E-05 | 2,81E-02 | 9,54E-02 | 2,17E-04 | 5,05E-02 |
| III/IV | 2,10E-03 | 2,13E-05 | 1,26E-02 | 3,88E-02 | 2,51E-04 | 3,19E-02 |
| White blood cell count (x10^9^/L) | | | | | | |
| < 20 | 1,88E-03 | 4,05E-05 | 2,57E-02 | 2,47E-02 | 1,63E-04 | 2,57E-02 |
| ≥ 20 | 4,84E-03 | 1,84E-05 | 3,10E-02 | 5,53E-02 | 3,00E-04 | 4,27E-02 |
| Lymphocytes (%) |  |  |  |  |  |  |
| < 75% | 2,95E-03 | 2,02E-05 | 3,22E-02 | 4,07E-02 | 3,12E-04 | 4,11E-02 |
| ≥ 75% | 4,92E-03 | 2,88E-05 | 2,87E-02 | 3,03E-02 | 2,24E-04 | 1,96E-02 |
| β_2_ M (mg/ml) |  |  |  |  |  |  |
| < 3 | 3,59E-03 | 1,95E-05 | 4,73E-02 | 8,47E-02 | 3,09E-04 | 6,84E-02 |
| ≥ 3 | 1,05E-02 | 1,58E-05 | 5,57E-02 | 2,01E-02 | 1,69E-04 | 1,79E-02 |
| LDH (UI/L) |  |  |  |  |  |  |
| < 450 | 3,54E-03 | 3,14E-05 | 1,58E-02 | 2,65E-02 | 3,19E-04 | 2,41E-02 |
| ≥ 450 | 7,09E-03 | 1,71E-05 | 1,21E-01 | 1,04E-01 | 1,79E-04 | 7,46E-02 |
| Hemoglobin (g/dl) |  |  |  |  |  |  |
| < 11 | 1,05E-02 | 1,95E-05 | 4,02E-02 | 1,24E-02 | 1,40E-04 | 1,44E-02 |
| ≥ 11 | 2,46E-03 | 2,54E-05 | 2,75E-02 | 5,48E-02 | 2,82E-04 | 4,37E-02 |

F: female; M: male; β_2_ M: beta2 microglobulin; LDH: lactate dehidrogenase
